# Supplementary material for: The High Proportion of Discordant EGFR Mutations among Multiple Lung Tumors
Source: Cancers (Basel). 2022 Jun 18;14(12):3011. doi: 10.3390/cancers14123011 (PMC9221401; doi:10.3390/cancers14123011)
Supplement: Supplementary file 1 [file cancers-14-03011-s001.zip › cancers-1735308-supplementary.pdf]

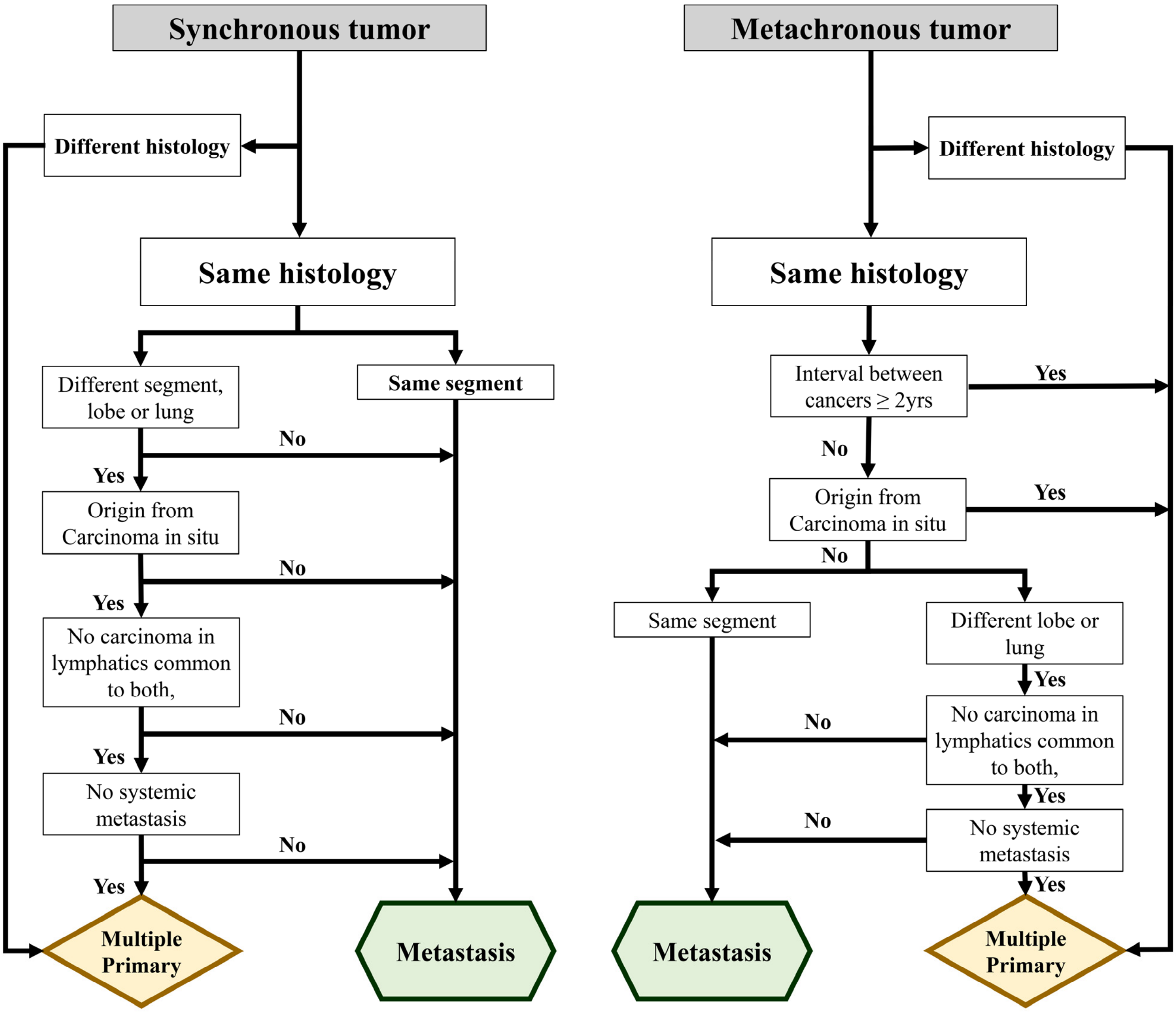

Figure S1. Algorithm for determining nature of multifocal lung cancer, adapted from Martini and Melamed criteria

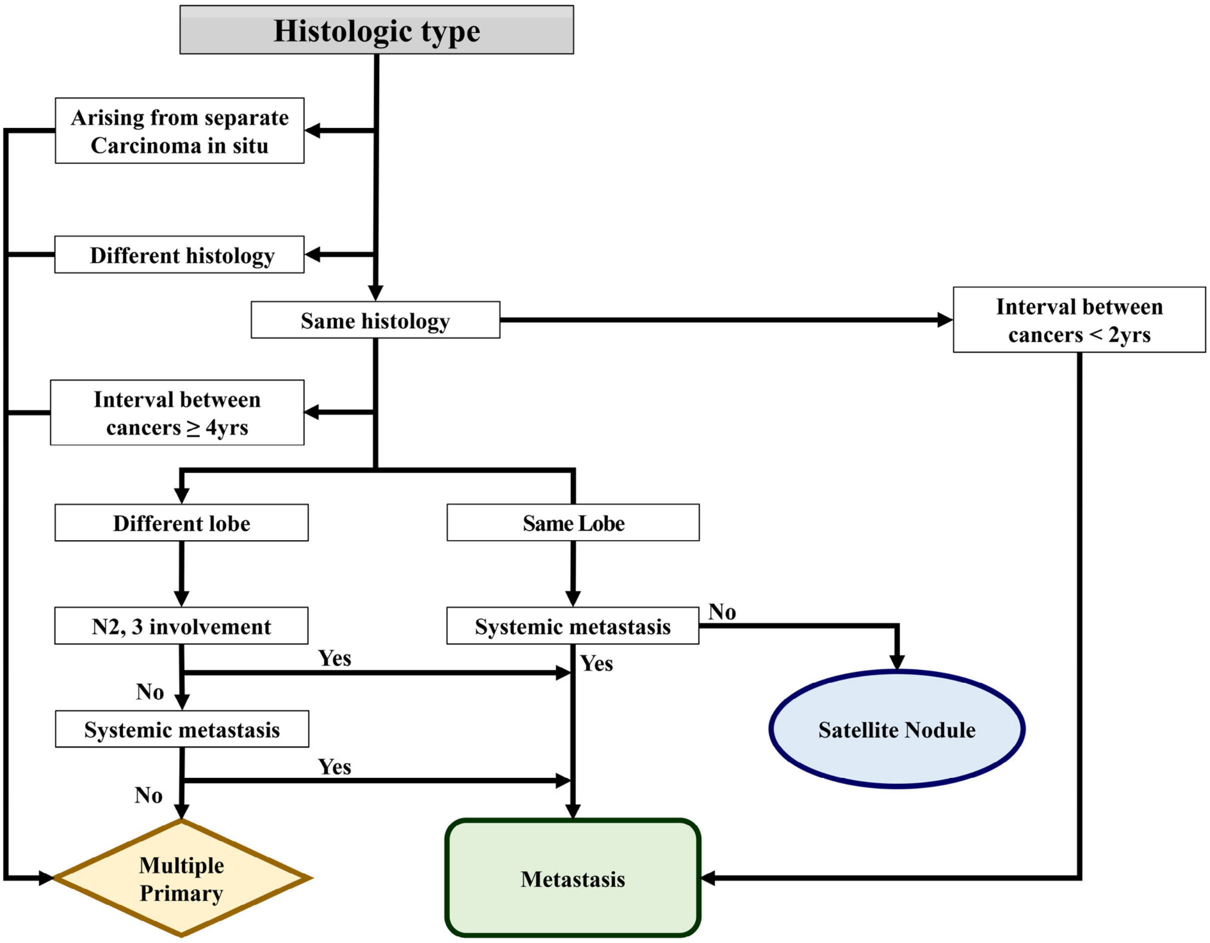

Figure S2 Algorithm for determining nature of multifocal lung cancer, adapted from the definition of satellite nodules, multifocal primary lung cancer, and metastasis, proposed by Americal College of Chest Physician

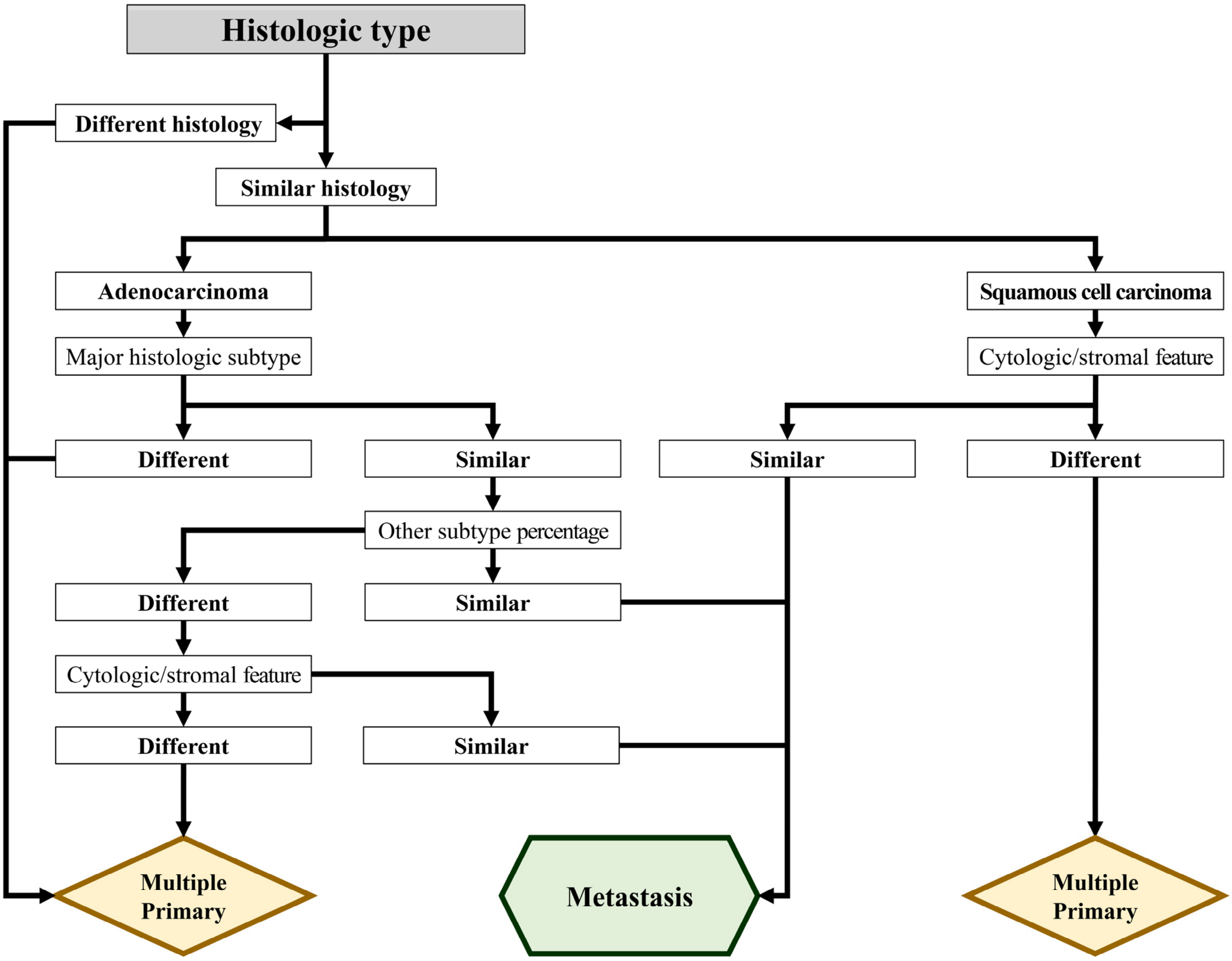

Figure S3. Algorithm for determining nature of multifocal lung cancer, adapted from Comprehensive histologic assessment methodology, proposed by Girard et al.

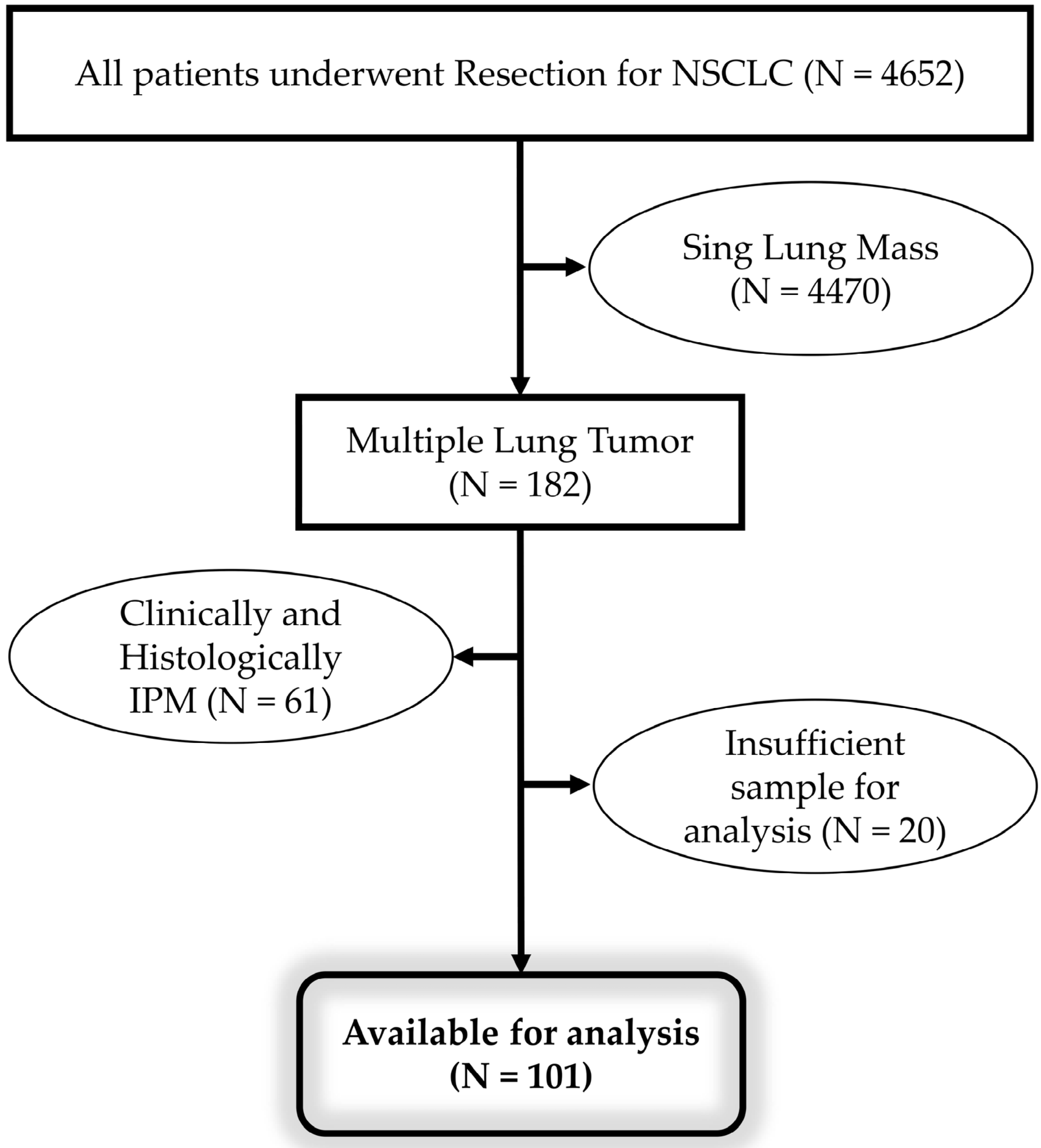

Figure S4. Workflow for patient selection.

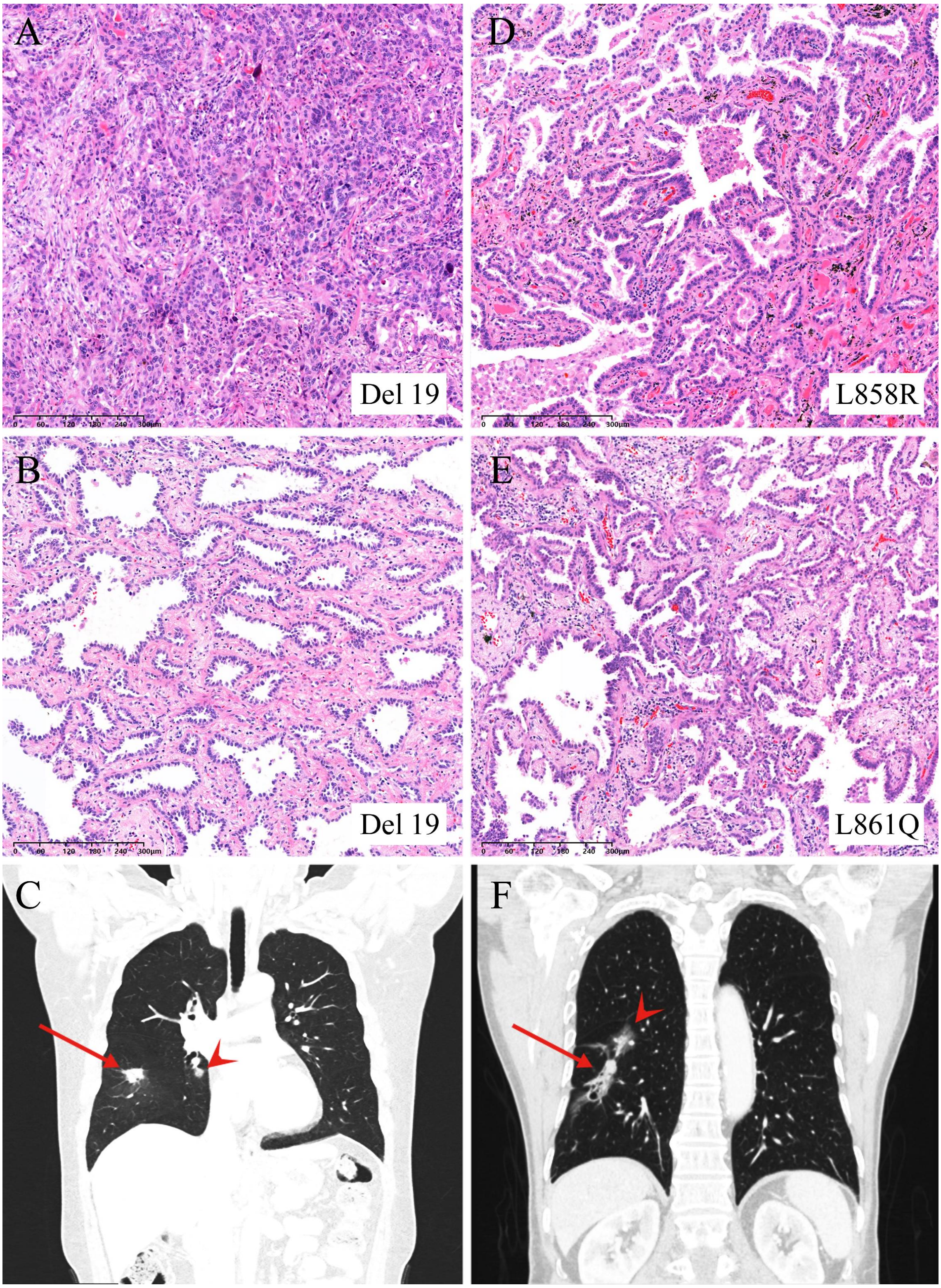

Figure S5. The examples of histologic feature and radiologic finding of adenocarcinomas with different histologic features (A-C) and same histologic features (D-F). The morphological subtypes of case 21 are different each other: (A) 80% Solid and 20% Acinar (x 40). (B) 50% Acinar, 30% Lepidic, and 20% Papillary. Both of tumors showed EGFR deletion mutation in exon 19. (C) Two tumors were located in same lobes [Arrow, tumor A; Arrowhead, tumor B]. On the other hands, case 75 showed same histology: (D) Papillary 60% and Acinar 40%. (E) Papillary 55% and Acinar 45%. (D) tumor had EGFR missense mutation in exon 21 (L858R) and (E) tumor also had EGFR missense mutation in different codon of exon 21 (L861Q). (F) Two tumors were located in different lobes [Arrow, tumor D; Arrowhead, tumor E].

Table S1. List of detectable EGFR mutation using cobas® EGFR mutation test v2

Exon	*EGFR* mutation group	*EGFR* Nucleic Acid Sequence
Exon 18	G719X	c. 2156G>C
		c. 2155G>A
		c. 2155G>T
Exon 19	Ex19Del	c. 2240_2251del12
		c. 2239_2247del9
		c. 2238_2255del18
		c. 2235_2249del15
		c. 2236_2250del15
		c. 2239_2253del15
		c. 2239_2256del18
		c. 2237_2254del18
		c. 2240_2254del15
		c. 2240_2257del18
		c. 2239_2248TTAAGAGAAG>C
		c. 2239_2251>C
		c. 2237_2255>T
		c. 2235_2255>AAT
		c. 2237_2252>T
		c. 2239_2258>CA
		c. 2239_2256>CAA
		c. 2237_2253>TTGCT
		c. 2238_2252>GCA
		c. 2238_2248>GC
		c. 2237_2251del15
		c. 2236_2253del18
		c. 2235_2248>AATTC
		c. 2235_2252>AAT
		c. 2235_2251>AATTC
		c. 2253_2276del24
		c. 2237_2257>TCT
		c. 2238_2252del15
		c. 2233_2247del15
Exon 20	S768I	c. 2303G>T
	T790M	c. 2369C>T
	Ex20Ins	c. 2307_2308ins9GCCAGCGTG
		c. 2319_2320insCAC
		c. 2310_2311insGGT
		c. 2311_2312ins9GCGTGGACA
		c. 2309_2310AC>CCAGCGTGGAT
Exon 21	L858R	c. 2573T>G
		c. 2573_2574TG>GT
	L861Q	c. 2582T>A

Table S2. Comparison of clinicopathological parameters between *EGFR*-wildtype tumors and *EGFR*-mutant tumors

Parameters	*EGFR*-wildtype (*n* = 95)	*EGFR*-mutant (*n* = 113)	*p*-value
Diagnosis			
Adenocarcinoma	71 (74.7)	111 (98.2)	< 0.001
Lepidic	18 (25.4)	13 (11.7)	0.017
Acinar	36 (50.6)	85 (76.6)	< 0.001
Papillary	9 (12.7)	10 (9.0)	0.43
Solid	6 (8.5)	2 (1.8)	0.058
Micropapillary	1 (1.4)	1 (0.9)	0.749
Cribriform	1 (1.4)	0 (0)	0.21
Squamous cell carcinoma	15 (15.8)	2 (1.8)	
Mucinous adenocarcinoma	7 (7.4)	0 (0)	
Large cell neuroendocrine carcinoma	1 (1.1)	0 (0)	
Adenosquamous carcinoma	1 (1.1)	0 (0)	
Tumor location			
Right	55 (57.9)	72 (63.7)	
Right upper lobe (RUL)	19 (20.0)	35 (31.0)	
Right middle lobe (RML)	8 (8.4)	14 (12.4)	
Right lower lobe (RLL)	28 (29.5)	23 (20.4)	
Left	40 (42.1)	41 (36.3)	
Left upper lobe (LUL)	21 (22.1)	11 (9.7)	
Left lower lobe (LLL)	19 (20.0)	30 (26.5)	0.84
Upper (RUL, RML and LUL)	46 (48.4)	79 (69.9)	
Lower (RLL and LLL)	49 (51.6)	34 (30.1)	0.002
T stage			
pT1mi	15 (15.8)	6 (5.3)	
pT1a	18 (18.9)	19 (16.8)	
pT1b	33 (34.7)	47 (41.6)	
pT1c	9 (9.5)	28 (24.8)	
pT2a	15 (15.8)	13 (9.7)	
pT2b	1 (1.1)	0 (0.0)	
pT3	2 (2.1)	0 (0.0)	
pT4	2 (1.1)	0 (0.0)	-
Tumor size			
≤ 3 cm	75 (78.9)	100 (88.5)	
> 3 cm	20 (21.1)	13 (11.5)	0.085
EGFR mutation			
No mutation	95 (45.7)	-	
Exon 19 deletion	-	40 (19.2)	
L858R	-	66 (31.7)	
L861Q	-	3 (1.4)	
G719X	-	1 (0.5)	
Exon 20 insertion	-	3 (1.4)	-

Data are presented as number of cases (%).

Table S3. Molecular alterations analyzed using next-generation sequencing and cobas® EGFR mutation test v2 in patients with *EGFR*-mutant/wildtype tumors

Case No.	*EGFR*-wildtype tumor			*EGFR*-mutant tumor		
	Location	Histologic diagnosis	Genetic alteration detected by NGS	Location	Histologic diagnosis	Genetic alteration detected by cobas
SMC009	Left lower lobe	Adenocarcinoma, Papillary predominant	*ERBB2* exon 20 Insertion, *CTNNB1* S33Y	Left upper lobe	Adenocarcinoma, Acinar predominant	L858R
SMC015	Left upper lobe	Adenocarcinoma, Acinar predominant	No mutation	Left upper lobe	Adenocarcinoma, Acinar predominant	L858R
SMC017	Left lower lobe	Adenocarcinoma, Papillary predominant	*ROS1* fusion	Right middle lobe	Adenocarcinoma, Acinar predominant	L858R
SMC043	Right lower lobe	Adenocarcinoma, Micropapillary predominant	*BRAF* V600E, *CTNNB1* S45P	Left upper lobe	Adenocarcinoma, Acinar predominant	Deletion in exon 19
SMC044	Right upper lobe	Adenocarcinoma, Acinar predominant	*CDK4* Amplification *MDM2* Amplification	Left lower lobe	Adenocarcinoma, Lepidic predominant	Deletion in exon 19
SMC076	Left lower lobe	Adenocarcinoma, Acinar predominant	*EGFR* A289V*	Left upper lobe	Adenocarcinoma, Acinar predominant	Insertion in exon 20
SMC078	Right lower lobe	Adenocarcinoma, Lepidic predominant	*FGFR* Amplification	Right upper lobe	Adenocarcinoma, Lepidic predominant	Deletion in exon 19
SMC085	Right lower lobe	Adenocarcinoma, Papillary predominant	*BRAF* V600E	Right upper lobe	Adenocarcinoma, Lepidic predominant	L858R
SMC100	Left lower lobe	Mucinous adenocarcinoma	*KRAS* Q61H	Left upper lobe	Adenocarcinoma, Lepidic predominant	L858R

*This variant is not included in the list of detectable mutation of cobas® EGFR mutation test v2

Table S4. Comparison of clinicopathological parameters between patients with molecular-concordant and molecular-discordant tumors

Parameter	Discordant (n = 60)	Concordant (n = 24)	p-value
EGFR mutation status			
EGFR-mutant/*EGFR*-mutant*	16 (26.7)	20 (83.3)	
EGFR-mutant/*EGFR*-wildtype	36 (60.0)	0 (0.0)	
EGFR-wildtype/*EGFR*-wildtype	8 (13.3)	4 (16.7)	-
Sex			
Female	36 (60.0)	19 (79.2)	
Male	24 (40.0)	5 (20.8)	0.095
Age (n, %) (years)			
< 65	26 (43.3)	11 (45.8)	
≥ 65	34 (56.7)	13 (54.2)	1.000
History of smoking			
No	42 (70.0)	20 (83.3)	
Yes	18 (30.0)	4 (16.7)	0.209
Present Smoking status			
Never-smoker	42 (70.0)	20 (83.3)	
Ex-smoker	13 (21.7)	3 (12.5)	
Current smoker	5 (8.3)	1 (4.2)	0.151
Tumor location			
Unilateral side, same lobe	17 (28.3)	11 (45.8)	
Same segment	11 (18.3)	8 (33.3)	
Other segment	6 (10.0)	3 (12.5)	
Unilateral side, different lobe	19 (31.7)	7 (29.2)	
Bilateral side	24 (40.0)	6 (25.0)	0.090
Largest Tumor size			
≤ 3 cm	51 (85.0)	20 (83.3)	
> 3 cm	9 (15.0)	4 (16.7)	0.849
Lymph node metastasis			
Absent	56 (93.3)	19 (79.2)	
Present	4 (6.7)	5 (20.8)	0.111
Histological pattern			
Concordant	35 (58.3)	13 (54.2)	
Discordant	25 (41.7)	11 (45.8)	0.727
Results of Martini and Melamed criteria[#]			
Multiple primaries	32 (53.3)	10 (41.7)	
Metastasis	28 (46.7)	14 (58.3)	0.469
Results of comprehesive histologic assessment[†]			
Multiple primaries	40 (66.7)	17 (70.8)	
Metastasis	20 (33.3)	7 (29.2)	0.712
Results of ACCP guideline[‡]			
Multiple primaries	34 (56.7)	19 (79.2)	
Metastasis or Satellite nodule	26 (43.3)	5 (20.8)	0.054

Abbreviation: ACCP, Americal College of Chest Physicians
Data are presented as number of patients (%).
*Two cases with rare EGFR mutations were not included in this category.
#The Martini and Melamed criteria were adapted from Martini et al. [11]
†The methodology of comprehensive histologic assessment was adapted from Girard et al. [14]
‡The guideline of ACCP was adapted from Alberts et al. [12] and Shen et al. [13]
